# Using an Inducible Promoter of a Gene Encoding *Penicillium verruculosum* Glucoamylase for Production of Enzyme Preparations with Enhanced Cellulase Performance

**DOI:** 10.1371/journal.pone.0170404

**Published:** 2017-01-20

**Authors:** Alexander G. Bulakhov, Pavel V. Volkov, Aleksandra M. Rozhkova, Alexander V. Gusakov, Vitaly A. Nemashkalov, Aidar D. Satrutdinov, Arkady P. Sinitsyn

**Affiliations:** 1 Federal Research Centre «Fundamentals of Biotechnology», Russian Academy of Sciences, Moscow, Russia; 2 Department of Chemistry, M.V.Lomonosov Moscow State University, Moscow, Russia; 3 G.K.Skryabin Institute of Biochemistry and Physiology of Microorganisms, Russian Academy of Sciences, Pushchino, Moscow region, Russia; National Renewable Energy Laboratory, UNITED STATES

## Abstract

**Background:**

*Penicillium verruculosum* is an efficient producer of highly active cellulase multienzyme system. One of the approaches for enhancing cellulase performance in hydrolysis of cellulosic substrates is to enrich the reaction system with β -glucosidase and/or accessory enzymes, such as lytic polysaccharide monooxygenases (LPMO) displaying a synergism with cellulases.

**Results:**

Genes *bglI*, encoding β-glucosidase from *Aspergillus niger* (AnBGL), and *eglIV*, encoding LPMO (formerly endoglucanase IV) from *Trichoderma reesei* (TrLPMO), were cloned and expressed by *P*. *verruculosum* B1-537 strain under the control of the inducible *gla1* gene promoter. Content of the heterologous AnBGL in the secreted multienzyme cocktails (hBGL1, hBGL2 and hBGL3) varied from 4 to 10% of the total protein, while the content of TrLPMO in the hLPMO sample was ~3%. The glucose yields in 48-h hydrolysis of Avicel and milled aspen wood by the hBGL1, hBGL2 and hBGL3 preparations increased by up to 99 and 80%, respectively, relative to control enzyme preparations without the heterologous AnBGL (at protein loading 5 mg/g substrate for all enzyme samples). The heterologous TrLPMO in the hLPMO preparation boosted the conversion of the lignocellulosic substrate by 10–43%; however, in hydrolysis of Avicel the hLPMO sample was less effective than the control preparations. The highest product yield in hydrolysis of aspen wood was obtained when the hBGL2 and hLPMO preparations were used at the ratio 1:1.

**Conclusions:**

The enzyme preparations produced by recombinant *P*. *verruculosum* strains, expressing the heterologous AnBGL or TrLPMO under the control of the *gla1* gene promoter in a starch-containing medium, proved to be more effective in hydrolysis of a lignocellulosic substrate than control enzyme preparations without the heterologous enzymes. The enzyme composition containing both AnBGL and TrLPMO demonstrated the highest performance in lignocellulose hydrolysis, providing a background for developing a fungal strain capable to express both heterologous enzymes simultaneously.

## Introduction

Filamentous fungi from the Ascomycota phylum proved to be efficient producers of highly active extracellular cellulase systems [1]. They include various species belonging to the genera *Trichoderma* (*T*. *reesei*, *T*. *longibrachiatum*, *T*. *harzianum*, *T*. *koningii*, etc.), *Penicillium* (*P*. *funiculosum*, *P*. *verruculosum*, *P*. *oxalicum*, *P*. *decumbens*, etc.), *Myceliophthora (M*. *thermophila*), *Chaetomium (C*. *thermophilum*) and others [2–5]. However, quite many fungal species produce multienzyme cocktails having a low level of the β-glucosidase (BGL) activity that is not enough for a fast conversion of cellobiose and other oligosaccharides, formed in cellulose hydrolysis under the action of cellulolytic enzymes (endoglucanases and cellobiohydrolases), to the final product, glucose [2, 6, 7]. Cellobiose released during the enzymatic hydrolysis of cellulose rather strongly inhibits cellobiohydrolases, thus reducing the cellulase system performance [8].

*P*. *verruculosum* B1-537 is a high-cellulase fungal strain that can also be used as a host to express homologous or heterologous enzymes [9, 10]. In spite of the high cellulase activity, B1-537 produces relatively low level of the BGL (~3% of the total secreted protein) that is not enough for efficient hydrolysis of cellulosic materials [10, 11]. On the other hand, it has been shown that extra addition of 40 units of the BGL from *Aspergillus niger* (AnBGL) to the *P*. *verruculosum* cellulase complex boosts the degree of cellulose conversion twice [10]. The boosting effect of BGL on the enzyme performance has also been reported for cellulases from *T*. *reesei* and other fungi [2, 12, 13].

Another approach for enhancing the hydrolytic potential of cellulases is adding a lytic polysaccharide monooxygenase (LPMO) to the reaction system [14, 15]. LPMOs represent a novel class of Cu-dependent enzymes that cleave cellulose and other polysaccharides via an oxidative mechanism, and they display a synergism with cellulases, acting as accessory enzymes (auxiliary activities) [14–16]. So, it is not surprising that modern commercial cellulase preparations of a new generation include LPMO in their composition [17].

Previously, we developed an expression system to produce homologous and heterologous enzymes in a host *P*. *verruculosum* B1-537 strain. It is based on an inducible promoter of the *cbhI* gene encoding cellobiohydrolase I (CBH I), a major cellulolytic enzyme of *P*. *verruculosum* [10, 18]. This inducible gene expression system leads to a significant increase in the level of a target protein expression, but the level of CBH I in the final enzyme preparations is often dramatically reduced. Using this approach, the F10 strain, a superproducer of the heterologous AnBGL comprising up to 80% of the total secreted protein, has been obtained [10]. LPMO from *T*. *reesei* (TrLPMO, formerly endoglucanase IV) has also been cloned and expressed in *P*. *verruculosum* B1-537 strain under the control of the *cbhI* gene promoter [19]. The content of the CBH I in the secreted multienzyme cocktail was significantly reduced, however the isolated recombinant TrLPMO, added to the basic *P*. *verruculosum* cellulase complex at the ratio 1:10, boosted the yield of glucose in cellulose hydrolysis almost twice, thus showing the great synergistic potential of the TrLPMO.

Recently, we found out a glucoamylase (GA) belonging to family 15 of glycoside hydrolases (GH15) in *P*. *verruculosum* [20], and then the *gla1* gene encoding GA was sequenced. Since glucoamylases catalyze the hydrolysis of starch and they are catalytically inactive toward cellulose, the regulatory parts of the *gla1* gene may be used for development of a new expression system that could be independently regulated by starch or starch derivatives, potentially preserving the high content of major cellulase enzymes in the secreted multienzyme cocktail. A starch-inducible expression system in *Acremonium cellulolyticus*, based on *glaA* promoter and terminator regions, has previously been developed by Inoue et al. [21] and successfully used for homologous expression of the CBH I (Cel7A) gene.

This article is focused on using the promoter part of the *gla1* gene for development of an expression cassette consisting of the *gla1* gene promoter fused to genes encoding AnBGL or TrLPMO, and testing the secreted multienzyme cocktails in hydrolysis of cellulosic substrates.

## Materials and Methods

### Fungal strains, fermentation media and enzyme preparations

*P*. *verruculosum* B1-537 strain [9, 10] was used as an auxotrophic host strain (*niaD-*) in transformation. The *Escherichia coli* MachI T1^®^ strain (Thermo Fisher Scientific Inc., Waltman, MA, USA) was used to obtain competent cells in the subcloning experiments.

The modified pUC19 vector (Thermo Fisher Scientific Inc., Waltman, MA, USA) was applied for cloning a full-length *gla1* gene including its promoter part. Thus, a plasmid pGA-GA was obtained.

The standard medium for cultivation of a recipient strain contained (g/L): cellulose– 40, yeast extract– 10, wheat bran– 10, KH_2_PO_4_−15, CaCl_2_−0.3, (NH_4_)_2_SO_4_−5.0. The same medium was used for obtaining the control enzyme preparation PvC2.

A medium for screening recombinant strains contained (g/L): cellulose– 40, wheat meal pretreated with a thermostable amylase– 200. The same medium was used for obtaining enzyme preparations containing AnBGL, TrLPMO and the control enzyme preparation (PvC1) without the heterologous enzymes.

### Construction of expression plasmids carrying *bglI* and *eglIV* genes encoding heterologous AnBGL and TrLPMO

PCR-product corresponding to the sequence of the *gla1* gene together with its promoter region (3924 bp, GenBank accession number: KY086000) was fused to a modified pUC19 linearized vector, containing *cbhI* terminator, by Ligation Independent Cloning method (LIC-method) [22]. The modified pUC19 vector, pUC-LIC, with specially designed 5`-and 3`-ends is a template for directional cloning of an expression cassette containing any heterologous or homologous genes with the coincident complementary 5`-ends [23].

Briefly, the PCR product (3924 bp) and linearized pUC-LIC vector were treated with T4 DNA polymerase (Thermo Fisher Scientific Inc., Waltman, MA, USA) in the presence dCTP and dGTP (Thermo Fisher Scientific Inc., Waltman, MA, USA), respectively. The treated insert was ligated into the treated pUC-LIC vector by mixing 50 ng of vector with 150 ng of insert. The mixture was incubated for 30 min at 22°C and then transformed into *E*. *coli* MachI competent cells using a standard transformation protocol [24]. Thus, a plasmid pGA-GA, containing the complete *gla1* gene with a promoter part, was obtained. The absence of mutations, additional insertions or deletions in the full-sized *gla1* cassette was confirmed by sequencing in both directions by the method described by Sanger et al. [25].

The resulting plasmid pGA-GA was subsequently used as a template for synthesis of the linear vector pGA. For this purpose, the following primers were generated:

pUC-gla-LIC5: TGCCGGCTGTGTTGAACGAAGGAAAAAAACAG
pUC-gla-LIC3: CCGGGCTTCTCCTCATAGACTTTCACTTTTTTCGACAG

Long PCR enzyme (Thermo Fisher Scientific Inc., Waltham, MA, USA) was used for amplification of the linear vector according to the following reaction conditions: 3 min at 95°C, followed by 20 cycles of 45 s at 95°C, 2 min at 50°C, 5 min at 68°C, and then 20 cycles of 10 min at 68°C, 10 min at 4°C. A product with a mass of about 5000 bp was purified by electrophoresis in 1% agarose gel in TBE buffer.

In order to amplify the *A*. *niger bglI* gene, the following primers were designed, based on information obtained from the *A*. *niger* genome database (DQ220304.1):

bglI-gla-LIC5: CAACACAGCCGGCATCATGAGGTTCACTTTGATCGAG
bglI-gla-LIC3: GAGGAGAAGCCCGGTTAGTGAACAGTAGGCAGAGACG

Amplification of the *A*. *niger bglI* gene was carried out by PCR using Long PCR enzyme mix (Thermo Fisher Scientific Inc., Waltham, MA, USA) under the following conditions: 5 min at 95°C, followed by 20 cycles of 1.5 min at 95°C, 2 min at 50°C, 2 min at 68°C, and then 20 cycles of 10 min at 68°C, 10 min at 4°C. The resulting PCR product was purified from agarose gel by QiAquick Gel Extraction Kit (QIAGEN, Valencia, CA, USA), and then it was cloned into the pGA linearized vector using ligation-independent cloning [22]. Thus, the plasmid construct pGA-BGL was obtained.

The same procedure was used to obtain the pGA-EGIV plasmid. The following primers were designed based on information obtained from the *T*. *reesei* genome database (XM_006961505.1):

eglIV-gla-LIC5: CAACACAGCCGGCATCATGATCCAGAAGCTTTCCAACCTC
eglIV-gla-LIC3: GAGGAGAAGCCCGGTCTAGTTAAGGCACTGGGCGTAGT

The pGA-BGL and pGA-EGIV expression plasmids were transformed into protoplasts of the host *P*. *verruculosum* B1-537 strain jointly with a transforming plasmid pSTA10 (10:1, μg), using the modified method described by Aleksenko et al. [26]. The pSTA10 plasmid contains a nitrate reductase gene providing complementation of a defective *niaD* gene in the host strain. This enables selection of the transformants on minimal media with 10 mM NaNO_3_. Transformation efficiencies typically reach 40–80 transformants per μg of transforming DNA, with co-transformation frequencies of 80% [26].

### Enzyme fractionation

Enzyme preparations were fractionated using a modified scheme described elsewhere [27]. In brief, proteins contained in a crude enzyme sample were preliminary precipitated with ammonium sulfate (80% saturation at 25°C) followed by a desalting procedure on a Bio-Gel P-4 (Bio-Rad Laboratories, Hercules, CA, USA) with the use of 0.02 M bis-Tris/HCl buffer, pH 6.8. Enzyme fractionation was carried out by anion-exchange chromatography on a Source 15Q HR 16/5 column (Pharmacia, Uppsala, Sweden). A sample containing 10 mg of protein was applied on the column equilibrated with 0.02 M bis-Tris/HCl buffer, pH 6.8. The bound protein was eluted with a gradient of 0 to 0.75 M NaCl at a flow rate of 1 mL/min (60 mL total volume). Protein concentration in collected fractions was determined by the modified Lowry method [28], using bovine serum albumin as the standard. Protein content in fractions containing the target heterologous enzymes (AnBGL or TrLPMO) was used to assay their content in the initial crude enzyme samples.

### MALDI-TOF mass spectrometry peptide fingerprinting

The in-gel tryptic digestion of protein bands after the SDS-PAGE was carried out essentially as described by Smith [29]. Trypsin (Promega, Madison, WI, USA, modified, 5 μg/mL) in 50 mM NH_4_HCO_3_ was used for a protein digestion. The resulting peptides were extracted from a gel with 20% aqueous acetonitrile containing 0.1% trifluoroacetic acid and subjected to MALDI-TOF mass spectrometry on an UltrafleXtreme TOF/TOF mass spectrometer (Bruker Daltonik GmbH, Bremen, Germany). Enzyme identification was carried out using Mascot peptide mass fingerprint server (http://www.matrixscience.com/).

### Enzyme activity assays

Glucoamylase activity was determined by analyzing reducing sugars released after 10 min of enzyme reaction with 5 mg/mL soluble starch from potato (Reakhim, Russia) at pH 4.7 (0.05 M Na-acetate buffer) and 30°C [30].

Avicelase activity was determined by analyzing reducing sugars released after 60 min of enzyme reaction with 5 mg/mL Avicel (microcrystalline cellulose from Vitek Company, Russia) at pH 5.0 (0.05 M Na-acetate buffer) and 40°C [31]. Enzyme activities against carboxymethylcellulose (CMC) and birchwood xylan (Sigma, St. Louis, MO, USA) were assayed at pH 5.0 and 50°C using a substrate concentration of 5 mg/mL [32]. Reducing sugars were analyzed by the Nelson-Somogyi method [33].

Enzyme activity against *p*-NP-β-glucopyranoside (*p*-NPG, Sigma, St. Louis, MO, USA) was determined at pH 5.0 and 40°C as described elsewhere [31]. Activity against cellobiose was determined at pH 5.0 and 40°C as described elsewhere [13].

Experiments on assaying enzyme activities were carried out in triplicates. Enzyme activities were expressed in international units. One unit of activity corresponded to the quantity of enzyme hydrolyzing 1 μmol of substrate or releasing 1 μmol of reducing sugars (in glucose equivalents) per minute.

### Hydrolysis of cellulosic substrates

Avicel (microcrystalline cellulose) was provided by Vitek Company (Russia). Aspen wood was pretreated by sequential two-stage milling on an impeller mill Mikrosilema IM-450 (Monolitstroy, Russia) with a rotor speed of 4500 rpm, productivity of 0.5–3.0 m^3^/h, 30 kW power, to obtain particles with an average size of 5–10 μm.

Hydrolysis of Avicel and milled aspen wood (100 mg/mL) by different enzyme preparations was carried out for 48 h at 50°C and pH 5.0 (0.1 M Na-acetate buffer). A weighed amount of each substrate (150 mg) was mixed with 1 mL of 0.1 M Na-acetate buffer (pH 5.0), containing 1 mM NaN_3_ to prevent microbial contamination, in a test tube (2 mL volume). Then, the tube was placed into a thermostated water bath, located on a magnetic stirrer, and 0.5 mL of suitably diluted enzyme solution in the same buffer was added to start the reaction. Hydrolysis was carried out under magnetic stirring. The enzyme loading in the reaction system was 5 mg of protein per 1 g of dry substrate (0.5 mg/mL). The enzyme preparation containing the heterologous TrLPMO was tested in Avicel hydrolysis also with an extra addition of 0.01 mg/mL purified cellobiose dehydrogenase (CDH) from *M*. *thermophila*, isolated as desribed elsewhere [15] (CDH was acted as an electron donor for LPMO). At definite time of the reaction, an aliquot of the suspension (0.1 mL) was taken, centrifuged for 3 min at 15,000 rpm, and the concentration of glucose in a supernatant was determined by the glucose oxidase method using Photoglucose kit from Impact Ltd. (Russia). Experiments were carried out in triplicates.

## Results and Discussion

### Cloning the *bglI* and *eglIV* genes

The *gla1* gene (GenBank accession number: KY086000) consists of 2032 bp including 3 introns. A promoter region takes 1889 bp. The classic TATA-box is located in -177 bp position upstream ATG-codon. The specific site of xylanase activator (XlnR) binding (GGCTAA) was found in -837 bp position. Canonical sites for binding the amylase activator (AmyR) were not found in a promoter region, but the CGGAAATTTGA sequence (-359 bp) could be assigned as a possible AmyR putative site [34]. This fact requires additional genetic studies since *P*. *verruculosum* is well known for its ability to produce large amounts of cellulases, which is critical for this microorganism, and the *gla1* gene promoter part might have a specific regulation of transcription mechanism.

**Fig 1:**
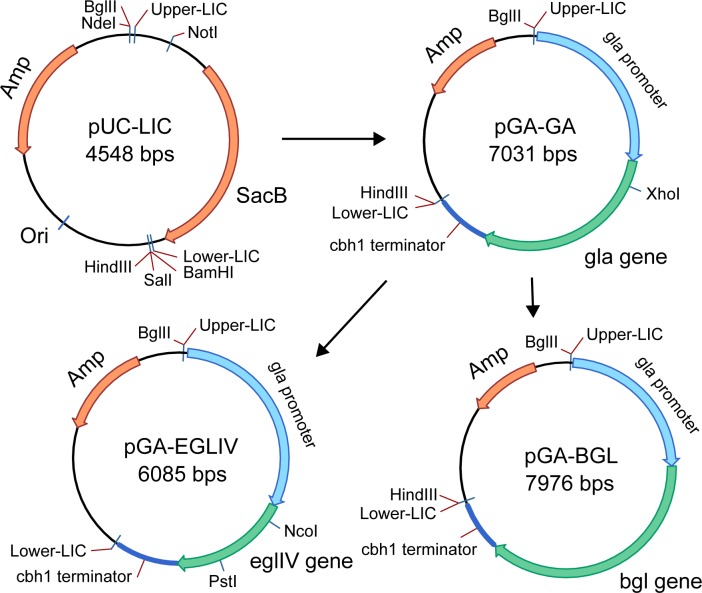
The scheme of cloning pGA-BGL and pGA-EGIV plasmids.

The pUC-LIC vector was linearized by BseRI with LIC-sites formation. The sticky 5`-ends in the linearized pUC-LIC vector and the full-sized *gla1* gene were obtained after T4-polymerase treatment with adding dGTP or dCTP, respectively. As a result of ligation, pGA-GA plasmid was developed (Fig 1). Then, new LIC-sites were added so that the new linearized pGA vector comprised a promoter region of the *gla1* gene and a terminator region of the *cbhI* gene. The *bglI* and *eglIV* genes encoding AnBGL and TrLPMO, respectively, were amplified and ligated to the pGA vector. So, it formed new pGA-BGL and pGA-EGIV plasmids where the *bgl1* and *eglIV* genes were controlled by a promoter part of the *gla1* gene. The scheme of cloning is shown in Fig 1.

### Obtaining enzyme preparations with heterologous AnBGL and TrLPMO

Nitrate reductase gene-defective recipient strain (*niaD-*) of *P*. *verruculosum* was co-transformed with the obtained pGA-BGL or pGA-EGIV plasmid together with the pSTA10 plasmid taken at a standard ratio (10:1, μg), using the transformation protocol described elsewhere [26]. The pSTA10 plasmid used in this study contains a nitrate reductase gene, allowing carrying out the selection of transformants in the medium with sodium nitrate. As a result, more than 200 transformants grown on the selective medium with sodium nitrate were obtained. Then PCR screening of the resulting colonies was carried out using a Phire Hot Start II DNA polymerase.

S1 Fig shows the results of PCR screening for the presence of the heterologous AnBGL and TrLPMO in the colonies. Selected transformants were cultured in Erlenmeyer flasks using a medium with wheat flour and Avicel. Fermentation was carried out for 6 days at 30°C and stirring mode 220 rpm. Enzyme activities against cellobiose, Avicel, birchwood xylan and synthetic *p*-NPG were measured in culture liquids. As can be seen from S1 Table, transformants numbered 6, 10 and 26 were the best BGL producers, since the specific BGL activity (against *p*-NPG) increased from 0.2 U/mg of protein (control) to 1.64–2.03 U/mg; the specific activity toward cellobiose also increased more than twice. It should be noted that along with the increase of cellobiase activity in the culture liquids of transformants, cellulase (Avicelase) and xylanase activities were not notably changed, but in some cases slightly increased. Therefore, the clones numbered 6, 10 and 26 were selected for obtaining enzyme preparations (named hBGL1, hBGL2 and hBGL3) for further testing.

Using similar screening methodology, one transformant expressing the highest amount of heterologous TrLPMO was selected, and the respective enzyme preparation, named hLPMO, was obtained using flask culturing.

**Fig 2:**
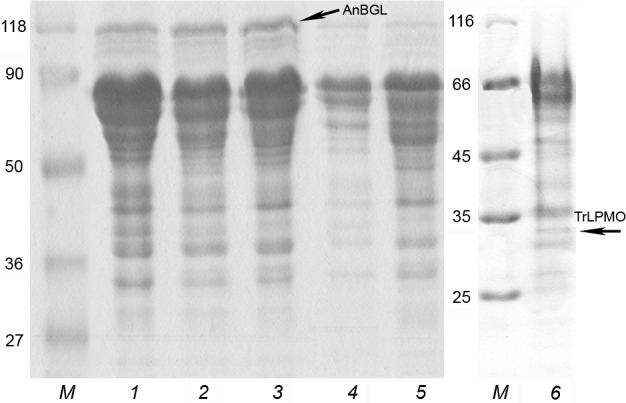
SDS-PAGE of *P. verruculosum* preparations. *M*, molecular markers (in kDa); *1*, hBGL1; *2*, hBGL2; *3*, hBGL3; *4*, PvC1; *5*, PvC2; *6*, hLPMO.

For comparison, a control *P*. *verruculosum* B1-537 strain was grown in a medium with wheat flour and in the standard medium with wheat bran and Avicel (the control preparations were named PvC1 and PvC2, respectively). Thus, six enzyme samples in total were obtained for testing, three of which contained the heterologous AnBGL and one–TrLPMO. The SDS-PAGE of the preparations is shown in Fig 2. Protein bands (~120 kDa and 33 kDa), corresponding to the heterologous enzymes, were identified by MALDI-TOF mass spectrometry peptide fingerprinting, and they are marked with arrows. The corresponding mass spectra are shown in S2 Fig, and the matching peptides are shown in S2 Table. Twenty six peaks matching by mass the specific tryptic peptides from the amino acid sequence of AnBGL were identified in the mass spectrum of a tryptic digest of the 120-kDa protein, and six peaks matching the tryptic peptides from TrLPMO were identified in the case of 33-kDa protein (S2 Fig, S2 Table). Unidentified peaks in the mass spectra belong to unspecific peptides or tryptic peptides carrying some modifications or peptides from impurity proteins, which could be present in the protein bands that were cut for a digestion with trypsin. The degree of coverage of AnBGL and TrLPMO amino acid sequences with identified peptides was 35 and 30%, respectively. It should be noted that a protein may be considered reliably identified by MALDI-TOF mass spectrometry if masses of not less than five peptides are matched and the sequence coverage is not less than 15% [35].

Table 1 shows specific activities of the enzyme preparations toward different substrates. The specific activity of hBGL1, hBGL2, hBGL3 and hLPMO preparations against soluble starch decreased relative to the PvC1 control preparation obtained in the same starch-containing medium. On the other hand, the specific BGL activity of the first 3 samples against cellobiose (and against *p*-NPG) increased up to 3.8-fold. The hLPMO sample demonstrated some decrease in cellulase (Avicelase and CMCase) activities in comparison with other enzyme preparations grown in a starch-containing medium, and also decrease in the BGL activity relative to the PvC1 sample.

**Table 1:** Specific activities (U/mg protein) of enzyme preparations.

Substrate	hBGL1	hBGL2	hBGL3	hLPMO	PvC1	PvC2
Soluble starch	0.27 ± 0.02	0.32 ± 0.02	0.50 ± 0.02	0.37 ± 0.03	0.57 ± 0.03	0.20 ± 0.02
Avicel	0.78 ± 0.04	0.82 ± 0.03	0.80 ± 0.04	0.64 ± 0.03	0.78 ± 0.03	0.80 ± 0.03
CMC	11.8 ± 0.5	10.6 ± 0.4	8.7 ± 0.4	8.2 ± 0.3	8.9 ± 0.4	17.1 ± 0.6
Xylan	42.1 ± 1.2	23.7 ± 0.9	23.8 ± 1.0	27.6 ± 1.1	26.5 ± 1.2	18.0 ± 0.9
Cellobiose	2.1 ± 0.1	5.0 ± 0.2	4.0 ± 0.2	0.8 ± 0.1	1.3 ± 0.1	0.9 ± 0.1
*p*-NPG	2.3 ± 0.1	4.2 ± 0.1	3.8 ± 0.2	1.0 ± 0.1	2.1 ± 0.1	1.1 ± 0.1

Using fractionation of the enzyme preparations by anion exchange chromatography followed by protein assay in the corresponding fractions containing heterologously expressed enzymes, the content of the AnBGL in hBGL1, hBGL2 and hBGL3 samples was estimated as 4, 10 and 8% of the total protein, respectively, while the content of TrLPMO in the hLPMO sample was found to be ~3%.

### Hydrolysis of cellulosic substrates

Avicel and milled aspen wood (100 mg/mL) were hydrolyzed for 48 h by the enzyme preparations under study. In each case, the protein concentration in the reaction system was 0.5 mg/mL (5 mg/g substrate).

**Fig 3:**
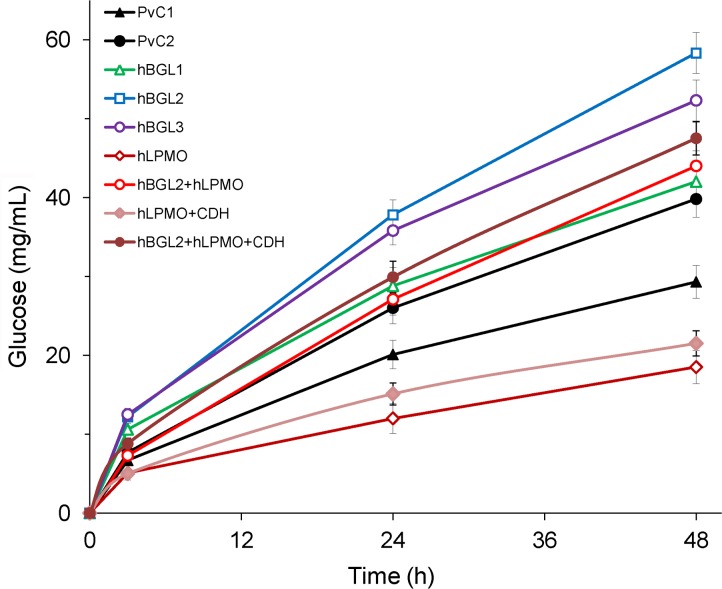
Progress kinetics of Avicel hydrolysis by different *P. verruculosum* preparations. Conditions: substrate concentration 100 mg/mL; protein loading 5 mg/g substrate; CDH loading (when applied) 0.1 mg/g substrate; 50°C; pH 5.0.

The hBGL2 preparation provided the maximum glucose yield (58.3 mg/mL) in Avicel hydrolysis (Fig 3). The hBGL1 and hBGL3 preparations provided the accumulation of 42.0 and 52.3 mg/mL of glucose in the reaction system after the same reaction time. Thus, the enzyme samples containing the heterologous AnBGL were more effective by 43–99% in Avicel hydrolysis relative to the PvC1 control preparation grown in the same starch-containing medium. They also demonstrated better performance (by 6–46%) in comparison with the PvC2 preparation grown in the standard medium for cultivation of a recipient strain. The hLPMO sample was less effective amongst all enzyme preparations tested. Obviously, the main reason for poor performance of this preparation was its lower cellulase (Avicelase and CMCase) and BGL specific activities (Table 1). Since cellobiohydrolases (CBH I and CBH II) are known to make a major contribution into Avicelase activity, it seems that their secretion by the respective recombinant fungal strain (hLPMO) was suppressed to some extent (up to 20% relative to other enzyme samples, taking into account their specific Avicelase activities). Since LMPOs need an electron donor for their function, the hLPMO preparation was also tested in Avicel hydrolysis with an addition of 0.01 mg/mL purified cellobiose dehydrogenase (CDH) from *M*. *thermophila* acting as an electron donor [15]. As expected, the CDH addition led to some increase in the glucose formation by the hLPMO sample, although its performance could not reach that of the PvC1 preparation (Fig 3). We also tested the hydrolytic performance of the hBGL2+hLPMO composition taken at the ratio 1:1, while maintaining the same protein loading (5 mg/g substrate) in the reaction system. This composition (both in the absence and in the presence of extra added CDH) provided better glucose yields (by 62 and 19%) than the control PvC1 and PvC2 preparations.

**Fig 4:**
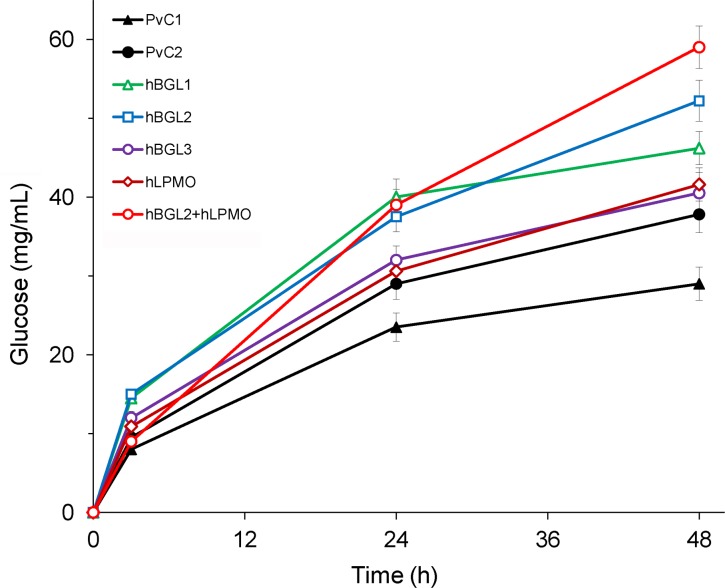
Progress kinetics of hydrolysis of pretreated aspen wood by different *P. verruculosum* preparations. Conditions: substrate concentration 100 mg/mL; protein loading 5 mg/g substrate; 50°C; pH 5.0.

The hydrolytic performance of the preparations under study was also tested on a pretreated lignocellulosic substrate (milled aspen wood, Fig 4). Compared to the PvC1 and PvC2 control preparations, the recombinant hBGL1, hBGL2 and hBGL3 samples provided higher glucose yields (by 40–80% and 7–38%, respectively) in 48-h hydrolysis. Higher rank of the hBGL1 preparation in aspen wood hydrolysis amongst those containing the AnBGL (when compared to its rank in Avicel hydrolysis, see Fig 3) may be explained by a higher xylanase activity of this sample (Table 1). Xylanase is known to act as an accessory enzyme to cellulases, providing not only the formation of sugars derived from xylan but also facilitating the access of cellulases to cellulose, thus enhancing the yield of glucose in hydrolysis of lignocellulosic substrates [36]. Obviously, the contribution of xylanases into boosting the cellulase activity of the hBGL1 sample was the highest in the first 24 h of the reaction, since at later stage of hydrolysis (24–48 h), when the remaining substrate became more resistant to a cellulase attack, the performance of this sample dropped more dramatically than that for the hBGL2 sample and hBGL2+hLPMO composition. The performance of the hLPMO sample in hydrolysis of the lignocellulosic substrate was approximately the same as that of the hBGL3, and it exceeded the performance of both PvC1 and PvC2 control preparations by 43 and 10% (Fig 4). Phenolic compounds, representing the building blocks of lignin, are known to act as electron donors for LPMOs [37, 38], so there was no need to add an external electron donor, such as CDH, to the reaction system. The highest glucose concentration after 48-h hydrolysis of aspen wood provided the hBGL2+hLPMO composition (1:1), the product yield being 103 and 56% higher than that for the control PvC1 and PvC2 preparations. These data indicate that the synergistic effect of two heterologously expressed enzymes (AnBGL and TrLPMO) on *P*. *verruculosum* cellulases is more important than the presence of only one of these components in the reaction system.

## Conclusions

The enzyme preparations produced by recombinant *P*. *verruculosum* strains, expressing the heterologous AnBGL or TrLPMO under the control of the *gla1* gene promoter in a starch-containing medium (pretreated wheat meal), proved to be more effective in hydrolysis of a lignocellulosic substrate than control enzyme preparations without the heterologous enzymes, including that grown in a standard medium optimized for cellulase production. At the same time, the enzyme sample containing TrLPMO (hLPMO) could not compete with the control preparations in hydrolysis of Avicel. The enzyme composition containing both AnBGL and TrLPMO demonstrated the highest performance in lignocellulose hydrolysis, providing a background for developing a fungal strain capable to express both heterologous enzymes simultaneously.

## Supporting Information

S1 Fig PCR screening of *P. verruculosum* fungal colonies for the presence of the heterologous AnBGL (A) and TrLPMO (B) by thermostable Pfire polymerase.
(TIF)

S2 Fig MALDI-TOF mass spectra of peptides derived from the in-gel tryptic digests of protein bands 120 kDa from hBGL2 sample (A) and 33 kDa from hLPMO sample (B) shown in Fig 2. Peaks matching by mass to specific tryptic peptides from AnBGL and TrLPMO, respectively, are marked.
(TIF)

S1 Table Activities of recombinant AnBGL clones in *P. verruculosum* culture liquids against natural and synthetic substrates (U/mg protein). (PDF)

S2 Table Peptide fingerprinting of the heterologously expressed AnBGL and TrLPMO using MALDI-TOF mass spectrometry of the in-gel tryptic digests of proteins (S2 Fig). (PDF)
