# Supplementary figures and images for: Using an Inducible Promoter of a Gene Encoding Penicillium verruculosum Glucoamylase for Production of Enzyme Preparations with Enhanced Cellulase Performance
Source: PLoS One. 2017 Jan 20;12(1):e0170404. doi: 10.1371/journal.pone.0170404 (PMC5249098; doi:10.1371/journal.pone.0170404)

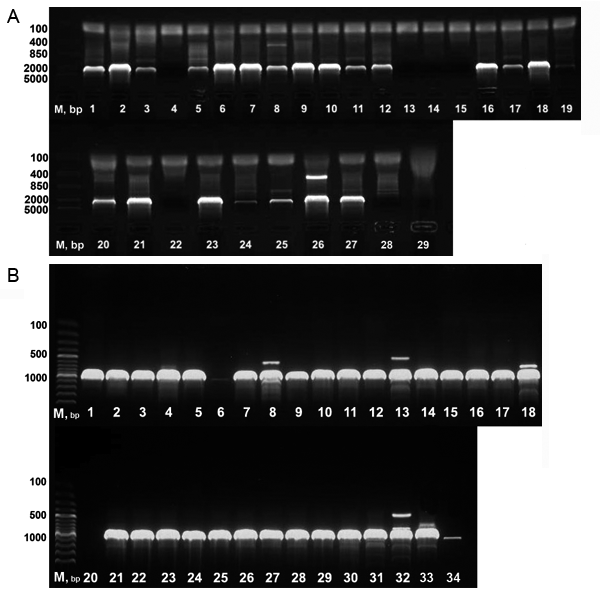

Supplement: S1 Fig — PCR screening of P. verruculosum fungal colonies for the presence of the heterologous AnBGL (A) and TrLPMO (B) by thermostable Pfire polymerase. (TIF) [file pone.0170404.s001.tif]

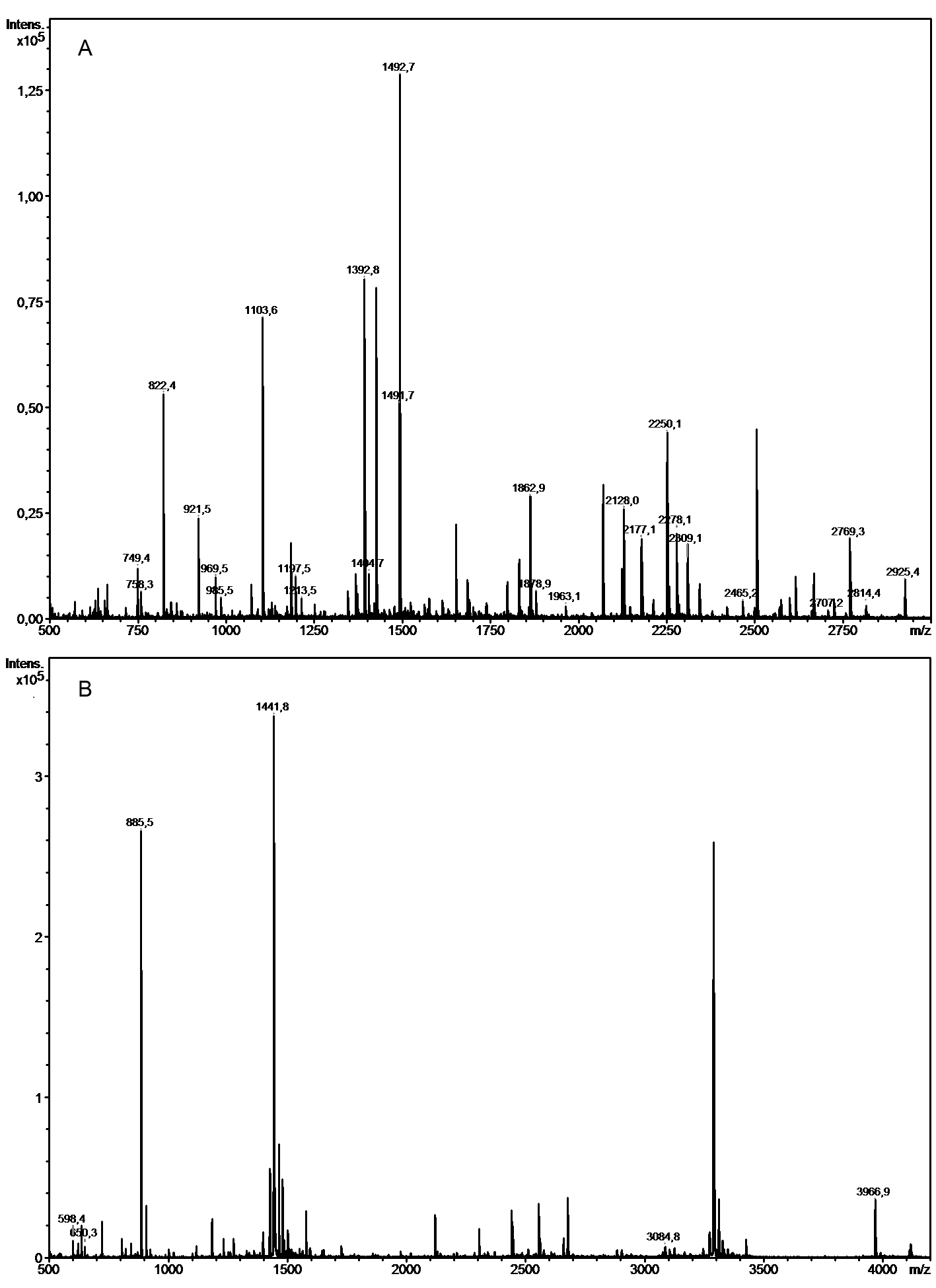

Supplement: S2 Fig — MALDI-TOF mass spectra of peptides derived from the in-gel tryptic digests of protein bands 120 kDa from hBGL2 sample (A) and 33 kDa from hLPMO sample (B) shown in Fig 2. Peaks matching by mass to specific tryptic peptides from AnBGL and TrLPMO, respectively, are marked. (TIF) [file pone.0170404.s002.tif]
