# Supplementary material for: Using an Inducible Promoter of a Gene Encoding Penicillium verruculosum Glucoamylase for Production of Enzyme Preparations with Enhanced Cellulase Performance
Source: PLoS One. 2017 Jan 20;12(1):e0170404. doi: 10.1371/journal.pone.0170404 (PMC5249098; doi:10.1371/journal.pone.0170404)
Supplement: S1 Table — (PDF) [file pone.0170404.s003.pdf]

S1 Table. Activities of recombinant AnBGL clones in *P. verruculosum* culture liquids against natural and synthetic substrates (U/mg protein).

Clones	Protein (mg/mL)	*p*-NPG	Cellobiose	Avicel	Xylan
1	5.23	0.32	3.9	1.49	22.6
2	5.77	0.95	4.4	1.50	32.5
3	5.78	0.83	4.3	1.33	27.0
5	5.64	1.17	4.3	1.51	28.4
**6**	6.02	**2.03**	**5.8**	1.43	26.7
7	6.12	1.35	5.1	1.43	33.1
8	5.72	1.07	4.3	1.42	25.5
9	5.77	0.73	4.2	1.36	27.9
**10**	5.83	**1.69**	**5.5**	1.41	30.6
11	5.69	0.53	4.0	1.53	29.2
12	5.65	0.8	4.3	1.40	30.7
16	5.81	0.51	4.1	1.48	29.7
17	5.67	0.74	4.2	1.48	29.0
18	5.95	0.77	4.3	1.37	30.3
20	5.41	0.32	4.0	1.34	22.6
21	5.70	0.98	4.5	1.28	25.8
23	6.07	0.93	4.4	1.39	32.8
25	5.59	0.43	3.9	1.10	19.6
**26**	5.54	**1.64**	**5.5**	1.12	24.6
27	5.40	0.70	3.8	1.31	19.5
28	5.3	0.59	3.9	1.29	18.7
B1-537	4.75	0.20	2.5	1.24	19.8
