# Supplementary material for: Using an Inducible Promoter of a Gene Encoding Penicillium verruculosum Glucoamylase for Production of Enzyme Preparations with Enhanced Cellulase Performance
Source: PLoS One. 2017 Jan 20;12(1):e0170404. doi: 10.1371/journal.pone.0170404 (PMC5249098; doi:10.1371/journal.pone.0170404)
Supplement: S2 Table — (PDF) [file pone.0170404.s004.pdf]

S2 Table. Peptide fingerprinting of the heterologously expressed AnBGL and TrLPMO using MALDI-TOF mass spectrometry of the in-gel tryptic digests of proteins (S2 Fig). The numeration of residues is given for mature proteins without signal peptides.

AnBGL			TrLPMO		
Peptide	Observed *m/z*	Calculated *m/z*	Peptide	Observed *m/z*	Calculated *m/z*
N100-R105	749.4	749.430	G65-K70	598.4	598.331
D342-K347	758.3	758.336	T258-R263	650.3	650.347
S577-K583	822.4	822.414	T107-K113	885.5	885.472
L739-R745	921.5	921.504	I144-R156	1441.8	1441.780
M821-R829	969.5	969.482	I114-K143	3084.8	3084.564
M821-R829[a]	985.5	985.477	A71-K106	3966.9	3966.883
N386-K395	985.5	985.531			
V359-R367	1103.6	1103.596			
G106-K116	1197.5	1197.520			
G106-K116[a]	1213.5	1213.515			
G117-R131	1392.8	1392.759			
A28-K40	1404.7	1404.740			
L330-R341	1491.7	1491.738			
H171-R181	1492.7	1492.697			
T205-R219	1862.9	1862.926			
T205-R219[a]	1878.9	1878.921			
L739-K755[b]	1963.1	1963.122			
D80-K99	2128.0	2127.962			
N456-K475	2177.1	2177.087			
L398-R419[c]	2250.1	2250.045			
N138-K158	2278.1	2278.139			
D802-K820	2309.1	2309.072			
R801-K820[b]	2465.2	2465.173			
Q780-R801[b]	2707.2	2707.380			
T476-R502	2769.3	2769.333			
V325-K347[b]	2814.4	2814.375			
T476-R503[b]	2925.4	2925.434			

[a] Oxidized methionine.

[b] Peptides containing missed cleavage(s) by trypsin.

[c] Acrylamide adduct on cysteine.
